# Image Mosaic Method Based on SIFT Features of Line Segment

**DOI:** 10.1155/2014/926312

**Published:** 2014-01-06

**Authors:** Jun Zhu, Mingwu Ren

**Affiliations:** School of Computer Science and Engineering, Nanjing University of Science and Technology, Nanjing 210094, China

## Abstract

This paper proposes a novel image mosaic method based on SIFT (Scale Invariant Feature Transform) feature of line segment, aiming to resolve incident scaling, rotation, changes in lighting condition, and so on between two images in the panoramic image mosaic process. This method firstly uses Harris corner detection operator to detect key points. Secondly, it constructs directed line segments, describes them with SIFT feature, and matches those directed segments to acquire rough point matching. Finally, Ransac method is used to eliminate wrong pairs in order to accomplish image mosaic. The results from experiment based on four pairs of images show that our method has strong robustness for resolution, lighting, rotation, and scaling.

## 1. Introduction

Recently image mosaic [1–4] has been an important subject in image processing researches. Image mosaic technologies hold extensive potential applications in remote sensing image processing, computer recognition, medical image analysis, artificial intelligence, and other fields. And also there are a number of techniques for capturing panoramic images of real world scenes [5]. Since, in real word application, the input images are taken at varying orientations and exposures, a feature-based registration technique similar to the pieces of literature [2, 6] is used to automatically align the input images. The image matching accuracy will have a direct influence on quality of panoramic image. Currently, there are two types of methods for image matching: one is the grayscale-based method that uses the correlation of grayscale in overlapping regions of two images to obtain optimal matching through correlation maximizing. The grayscale-based method is easy to implement, but it is relatively sensitive to grayscale changes in images, especially under variable lighting. The other matching methods based on image features use image pixel values to extract features. Because these features are partially invariant to lighting changes, matching ambiguity would be excellently resolved in the process of image matching. As for the extraction of image feature points, there already have been many proved methods, for example, Harris method [3], Susan method [7], and Shi-Tomasi method [8]. This feature-based image mosaic method has two main advantages as follows: (1) the computation complexity of image matching will be significantly reduced for the reason that the image feature points are far less than pixels; (2) the feature points have strong robustness for unbalance lighting and noises; as a result, the quality of image mosaic would be improved.

**Figure 1:**
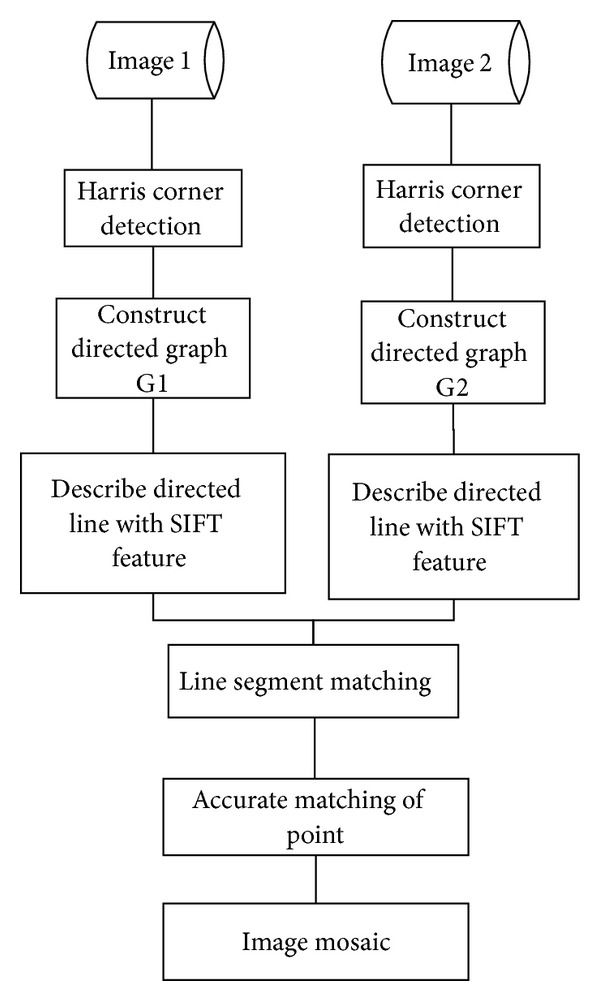
Flowchart of our method.

The methods to describe point features are mainly dependent on the description of image blocks [9], such as SIFT (Scale Invariant Feature Transform) method [10]. The pieces of literature in recent years [11–14] indicated that the researchers attached more and more importance to the improvement of SIFT-based matching accuracy while limiting the computation volume. The method of image feature description plays a vital role in the quality of image mosaic. And the performance is evaluated by robustness and speed. There have been several researches on how to improve the robustness and reduce the computation time [11, 15]. Inspired by the pieces of literature [16–18], we have known that the mesh feature of images has relatively strong robustness for image rotation and scaling. This paper proposes a matching method based on SIFT features of directed line segment in images. In order to improve the robustness and efficiency, similar to the pieces of literature [19, 20], we set our method as follows. It firstly uses Harris corner detection operator to extract key points and then constructs directed graph of extracted points. Secondly, it describes directed line segments with SIFT feature and matches them to attain rough matching of points. Finally, it adjusts matching points and eliminates wrong pairs through Ransac method to accomplish collage of images. The whole framework of our method can be seen from Figure 1. The method proposed here has the following major advantages: (1) the description based on features of mesh has strong robustness for image rotation, distortion, and scaling; (2) the description of directed line segment with SIFT features has certain robustness for image lighting influences and rotation; (3) rough point matching by statistical method could improve matching accuracy.

The remainder of our paper is structured as follows.  Section 2 reviews the Harris method and SIFT feature description.  Section 3 describes our novel image mosaic method based on SIFT features of line segment. Experiments and analysis are demonstrated in Section 4. Conclusions are made in Section 5.

## 2. Harris Corner Detection and SIFT Feature Description 

When SIFT method is adopted to detect feature points, the computation in the procedure of image pyramid construction, key point location determination by extreme value detection and others, will be of very time consuming. With the advantages of good performance of Harris operator to detect corner points, and so the combination of feature points detection by Harris method and SIFT description, the image mosaic will be accelerated.

### 2.1. Harris Corner Detection

Harris operator is a sort of signal-based point feature extraction operator proposed by Chris Harris [21] that is characterized with simple computation, homogeneous and reasonable extracted corner features, available quantitative extraction, and stable operator. Harris corner detection method applies the self-correlation function theory for signal processing that defines the point with both high row curvature and high line curvature. Harris method can be expressed as

(1) $$\begin{array}{c} M=G\left(\tilde{s}\right)⊗\left(\begin{array}{cc} g_{x}^{2} & g_{x}g_{y}\\ g_{x}g_{y} & g_{y}^{2} \end{array}\right), \end{array}$$

in which *g*_*x*_ means the gradient at *x* direction, *g*_*y*_ means the gradient at *y* direction, $G(\tilde{s})$ means the Gaussian template, and ⊗ means the convolution between Gaussian template and the function.

On this basis, the response function of Harris corner detection method is

(2) $$\begin{array}{c} R=\det\left(M\right)-k{\left(\text{trace}\;\left(M\right)\right)}^{2}, \end{array}$$

in which det(*M*) means the matrix determinant, trace (*M*) means the matrix trace, and *k* means a default constant, which generally is 0.04*∼*0.06. In practical application, a point will be determined to be a corner point if its corner response function is greater than the given threshold value *T*. Different images have very largely different structures and textural features, which leads to their appropriate threshold value *T* which will differ in a large range. Later, Shi and Tomasi brought forward an improved method: if the smaller between two eigenvalues is greater than the minimum threshold value, it will get strong corners. The method proposed by Shi and Tomasi is relatively perfect and could obtain better results under many conditions.

### 2.2. SIFT Feature Description

SIFT [10] extracts invariant feature based on invariant descriptor that was proposed by Lowe in 2004. SIFT feature fundamentally remains invariant to image translation, rotation, scaling, brightness variation, and noises. SIFT feature description mainly includes two steps: (1) determine direction parameter of feature points; (2) use graphic information around feature points to construct 128-dimensional descriptor.

#### 2.2.1. The Determination of Direction Parameter

To ensure rotated invariance of descriptor of feature points, it shall calculate the main direction of feature points and create SIFT feature descriptor at this main direction. For the detected feature points, finite difference calculation will be applied to figure out pixel gradient module *m* and angle of gradient amplitude *θ* in the region with the feature point as center. The formalists are as follows:

(3) $$\begin{array}{c} m\left(x,y\right)=\sqrt{L_{1}^{2}+L_{2}^{2}},\\ L_{1}=L\left(x+1,y\right)-L\left(x-1,y\right),\\ L_{2}=L\left(x,y+1\right)-L\left(x,y-1\right),\\ \theta \left(x,y\right)=\arctan\left(\frac{L\left(x,y+1\right)-L\left(x,y-1\right)}{L\left(x+1,y\right)-L\left(x-1,y\right)}\right), \end{array}$$

in which *L*(*x*, *y*) means pyramid image grayscale of the feature point at (*x*, *y*) on its scale. Then use histogram to statistically state pixel gradient module and direction in this region. The abscissa axis of histogram is the angle of amplitude of gradient direction, and the ordinate axis is the accumulated value of gradient module corresponding to gradient direction angle. The graphic of gradient direction is divided into 36 columns according to the range of 0°~360° that is each 10° is for a column. The peak value of histogram represents the direction of image gradient in neighborhood of this feature point, which is the main direction of this point, and selects 80% of peak value as auxiliary direction value. Therefore, one feature point could be set with many directions to enhance the robustness of matching.

#### 2.2.2. The Construction of SIFT Descriptor

Rotate local region around feature point by *θ*; the angle is represented by the main direction to maintain its rotational invariance. In the rotated region, equally divide the 16 × 16 rectangular window with the feature point as center into 4 × 4 subregions, and in each subregion, figure out gradient histogram of eight directions (0°, 45°, 90°, 135°, 180°, 225°, 270°, and 315°). In the same way, it is necessary to conduct Gaussian weighting processing to each pixel's gradient magnitude. Therefore, each feature point will generate a 128-dimensional eigenvector.

## 3. Directed Line Segment Matching

The description method based on features of line segment not only can acquire local information of images, such as textures and gradients, but also can be able to obtain image content between line segments and other information. Our method has two creative aspects: (1) it describes image features through the description of connecting line between key points, not through image blocks; (2) the description method based on line segment can reflect topological structures of image and therefore it has relatively high robustness for nonlinearly distorted and rotated images.

### 3.1. Line Segment Features

Given two images *I* and *I*′ to be matched, we use Harris operator to detect key points in these two images and utilize detected key points to construct two directed graphics, *G* = (*V*, *E*) and *G*′ = (*V*′, *E*′). Define *V* = {*a*_1_, *a*_2_,…, *a*_*n*_} and *V*′ = {*b*_1_, *b*_2_,…, *b*_*m*_} that mean key points extracted from images *I* and *I*′, respectively, and *E* and *E*′ are the edge sets of directed graphics *G* and *G*′, respectively, where *E* = {(*a*_*i*_, *a*_*j*_),  *i* ≠ *j*}, *E*′ = {(*b*_*i*_, *b*_*j*_),  *i* ≠ *j*}. Take the directed line segment between two key points as the object of feature description. Set one edge of graph *G*, *e*_*ij*_ ∈ *E* (the starting point is *a*_*i*_, and the end point is *a*_*j*_). In order to reduce computation time of describing features of the edge *e*_*ij*_, we equidistantly sample five points from the edge *e*_*ij*_, {*p*_1_, *p*_2_, *p*_3_, *p*_4_, *p*_5_}, in which *p*_*k*_ = *p*_*i*_ + ((*k* − 1)/4)(*p*_*j*_ − *p*_*i*_), *k* = 1,…, 5; *p*_*i*_ refers to the image coordinate of point *a*_*i*_. As to the feature points {*p*_1,_ *p*_2_, *p*_3_, *p*_4_, *p*_5_} sampled from the directed line segment *e*_*ij*_, extract their SIFT feature, respectively, *S* = (*s*_1_, *s*_2_, *s*_3_, *s*_4_, *s*_5_). Each column in *S*, *s*_*k*_, *k* = 1,2,…, 5, is a 128-dimensional vector, which represents SIFT feature of point *p*_*k*_. Those feature points sampled uniformly from line segment have strong robustness for image scaling, rotation, and changes in resolution.

### 3.2. Nearest-Neighbor Matching of Line Segments

With the purpose of rough matching, a line segment-matching method based on nearest-neighbor criterion is proposed. It is assumed that image *I* has *n*_1_ directed line segments, *L* = [*l*_1_, *l*_2_,…, *l*_*n*_1__], and image *I*′ has *n*_2_ directed line segments, *L*′ = [*l*_1_′, *l*_2_′,…, *l*_*n*_1__′], and a static matrix, *K* ∈ *R* ^*n*_1_×*n*_2_^, could be defined as

(4) $$\begin{array}{c} K\left(i,j\right)=\{\begin{array}{cc} 1 & l_{j}^{\prime }\;\text{is}\;\;\text{the}\;\;\text{nearest}\;\;\text{neighbor}\;\;\text{points}\;\;\text{of}\;\;l_{i}\\ 0 & \text{otherwise}. \end{array} \end{array}$$

Because this paper uses a matrix to describe features of line segments, it takes *F*-norm of the matrix as measurement in computation of nearest-neighbor points; that is, *d*(*l*_*i*_, *l*_*j*_′) = ||*S*_*i*_−*S*_*j*_′||_*F*_ and *S*_*i*_ means features of line segment *l*_*i*_; *S*_*j*_′ means features of line segment *l*_*j*_′. Computation procedure of matrix *K* is as shown in Procedure 1.

### 3.3. Matching of Points

In last section, we obtain matching of directed line segments through the rule of nearest-neighborhood. It is necessary to get more accuracy of point matching to exact mosaic of image. With the sets of key points in two given images, *V* = {*a*_1_, *a*_2_,…, *a*_*n*_} and *V*′ = {*b*_1_, *b*_2_,…, *b*_*m*_}, we use the method based on statistical voting to obtain the frequency of point matching. As we know, if two straight lines match each other, their starting point and end point should match each other. In the first place, initiate a statistical matrix *G* ∈ *R* ^*n*×*m*^ into null matrix; the computation procedure of *G* is as shown in Procedure 2. In matrix *G*, the larger element value indicates higher probability for corresponding point matching.

The criteria to select matching points are:

1. *G*(*i*, *j*) > *σ*, in which *σ* is a proper positive number;
2. selecting the point corresponding to maximum elements in each row and each column as the matching point, *G*(*i*, *j*) > *σ*, in which *σ* is a proper positive number, such as in *G*(*i*, *j*), if the elements in row *i* and column *j* are maximum, *a*_*i*_ and *b*_*j*_ match each other; it will set all elements in row *i* and column *j* to be null;
3. if the maximum element in row *i* and the maximum element in column *j* are not the same, it will randomly select one of them; select for example the maximum element in row *i*, *G*(*i*, *q*), *a*_*i*_ and *b*_*q*_ match each othering; it will set all elements in row *i* and column *q* to be null.

## 4. Experiment and Analysis

**Figure 2:**
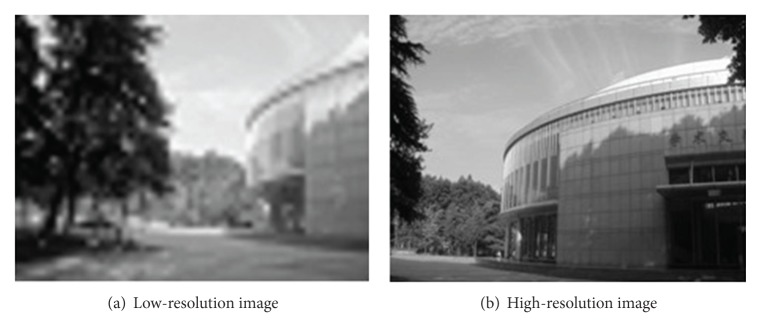
Images with different resolutions.

The experiments select four pairs of images taken by ordinary camera. In order to prove the effectiveness of our method, the selected four pairs of images vary largely in lighting, rotation, scaling, and resolution. In Figure 2, the left image and the right image are different in resolution.  Figure 7 shows that the objects in the two images are different in orientation. The two images in Figure 12 are taken under different lighting conditions, and the left one is exposed more time. Moreover, in Figure 17, the building in the left image is larger than the one in the right image. All images to be matched in the experiment are with the size of 461 × 346 in pixel.

### 4.1. Experiment on Images with Different Resolutions

**Figure 3:**
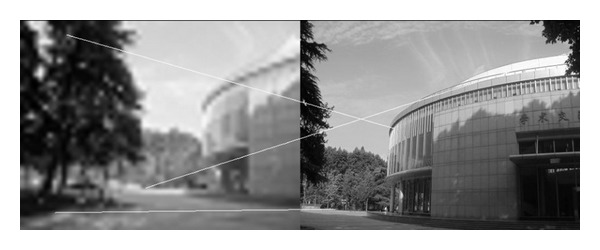
Results of Harris corner matching based on grayscale feature.

**Figure 4:**
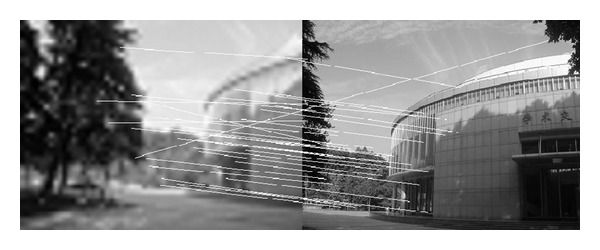
Results of matching based on SIFT feature.

**Figure 5:**
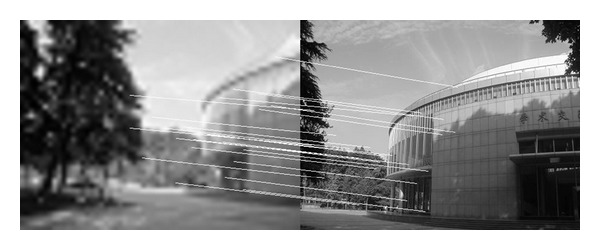
Results of matching by the method proposed in our paper.

**Figure 6:**
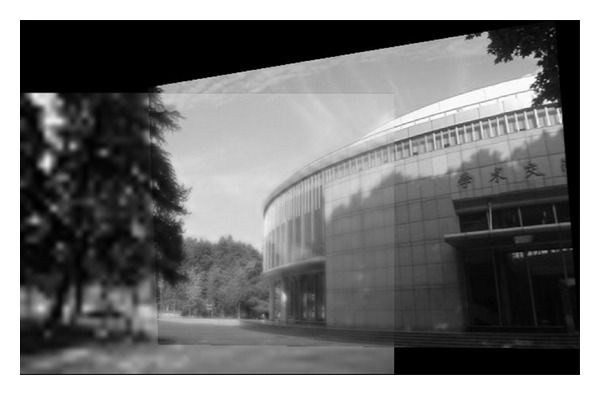
Mosaic results by the method proposed in this paper.

In this experiment, we choose two images taken by an ordinary camera. The two images are preprocessed that the two images have different resolutions.  Figure 2(a) is a low-resolution image, so it looks blurred. In addition, Figure 2(b) looks more clean since it has high resolution. Figures 3–5, show the matching results from different methods.  Figure 6 gives the last panoramic image stitched by our method.

### 4.2. Experiment on Rotated Images

**Figure 7:**
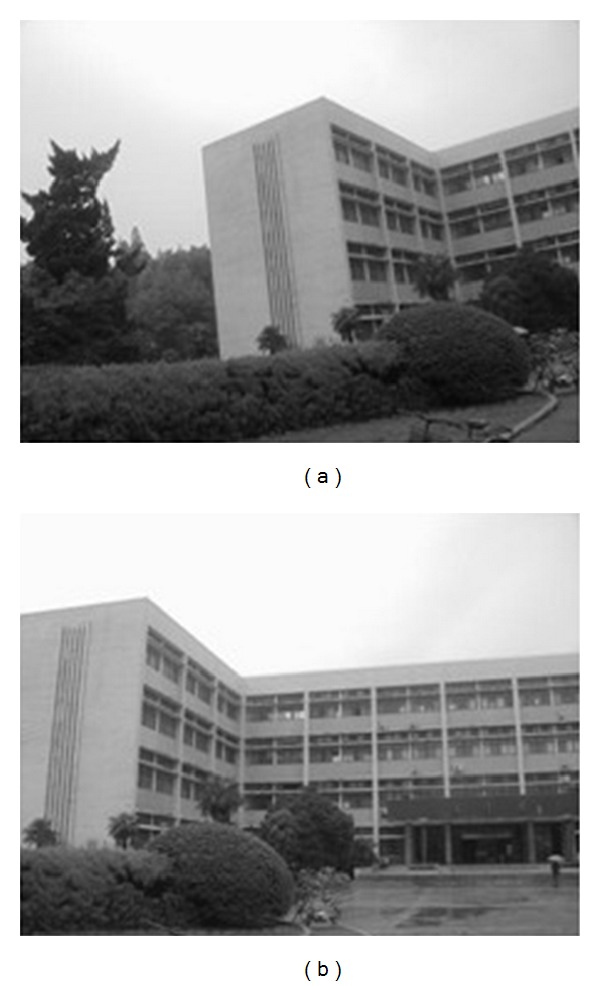
Object-rotated image; the building in both (a) and (b) is rotated.

**Figure 8:**
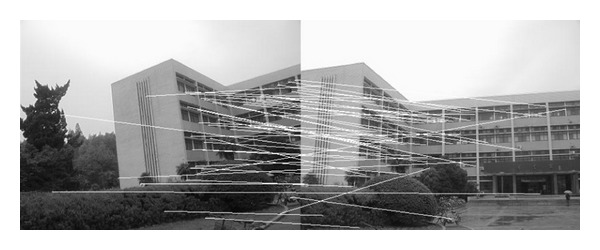
Results of Harris corner matching based on grayscale feature.

**Figure 9:**
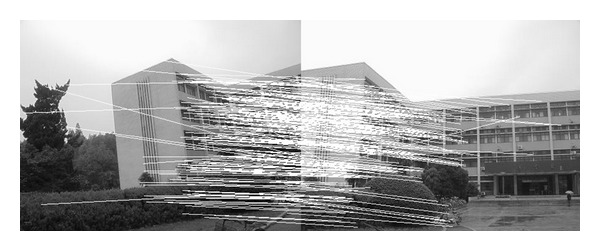
Results of matching based on SIFT feature.

**Figure 10:**
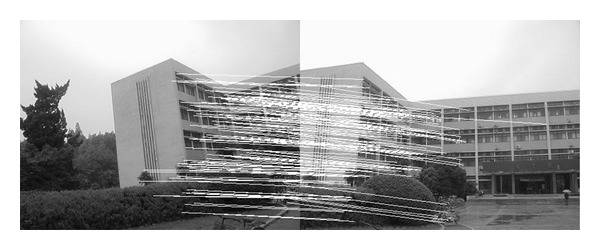
Results of matching by the method proposed in this paper.

**Figure 11:**
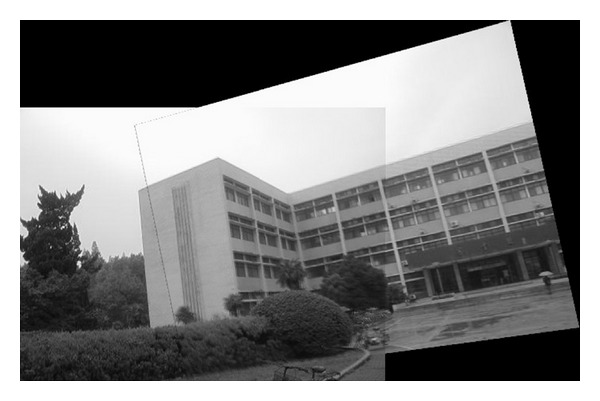
Mosaic results by the method proposed in this paper.

In this experiment, the two images to be stitched in Figure 7 were taken by ordinary camera. A building has different orientations in the two images because the position of camera was changed. Results of matching by different methods are shown in Figures 8–10. Moreover, Figure 11 gives the last panoramic image stitched by our method.

### 4.3. Experiments on Different Lighting Condition Images

**Figure 12:**
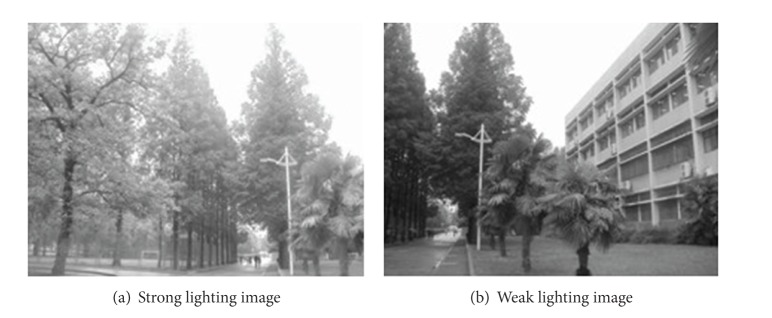
Different lighting images.

**Figure 13:**
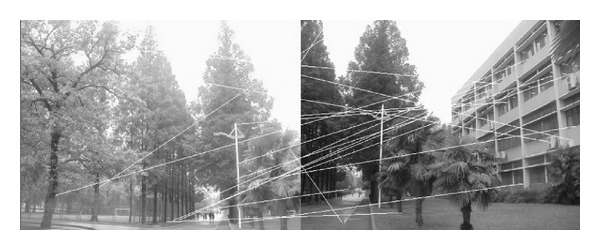
Results of Harris corner matching based on grayscale feature.

**Figure 14:**
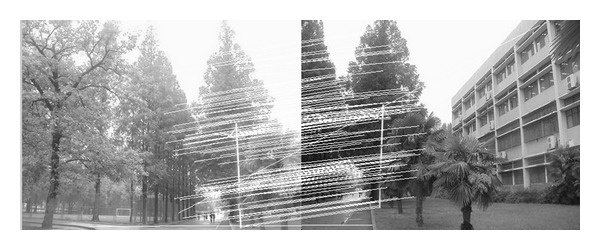
Results of matching based on SIFT feature.

**Figure 15:**
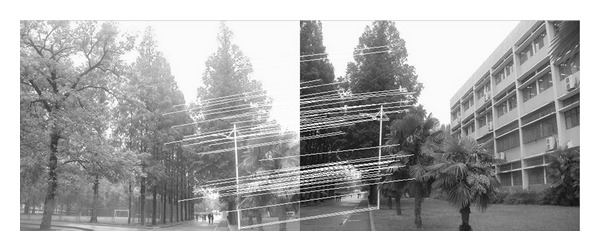
Results of matching by the method proposed in this paper.

**Figure 16:**
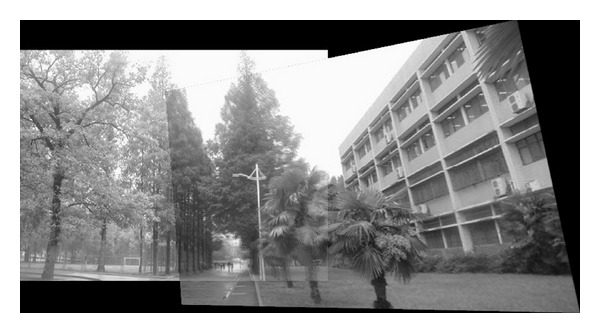
Mosaic results by the method proposed in this paper.

In this experiment, we choose two images sampled from original camera. The lighting conditions in the two images are largely different.  Figure 12(a) has longer exposure time. Figures 13–15 are the results of matching by different methods. And Figure 16 gives the panoramic image stitched by our method.

### 4.4. Experiment on Object Scaling Images

**Figure 17:**
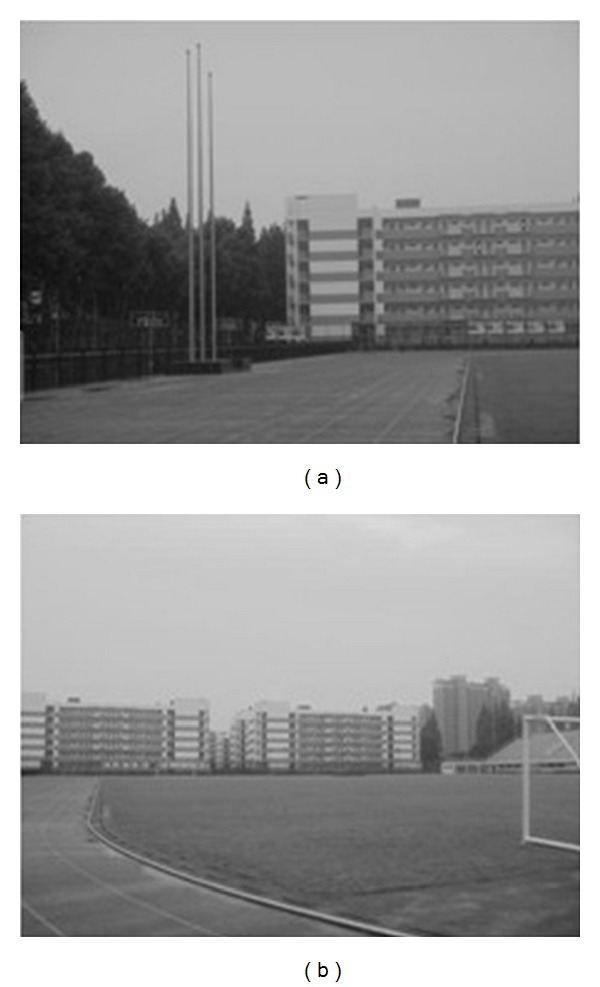
Different scale images; the building in (a) is enlarged compared with the one in (b).

**Figure 18:**
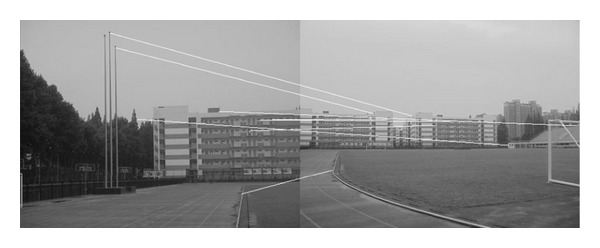
Results of Harris corner matching based on grayscale feature.

**Figure 19:**
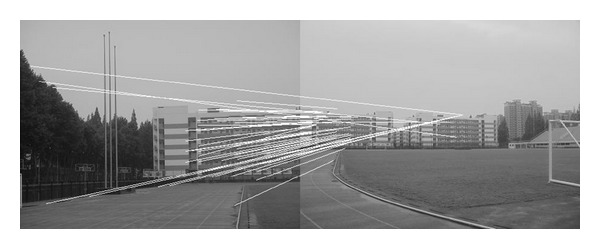
Results of matching based on SIFT feature.

**Figure 20:**
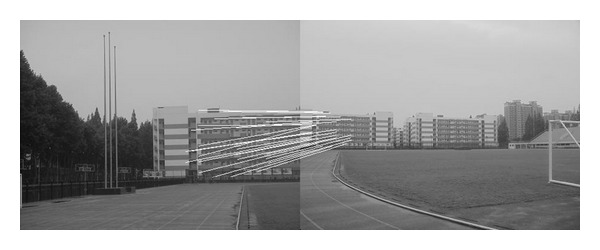
Results of matching by the method proposed in this paper.

**Figure 21:**
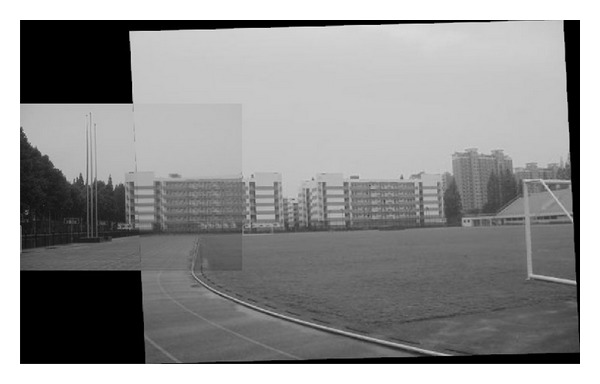
Mosaic results by the method proposed in this paper.

In this experiment, we choose two images that have different scale. The same building in the two images has different scales.  Figure 17(a) is taken when the lens of camera is zoomed relative to Figure 17(b). Therefore, the building in the left image is larger than the one in the right. Figures 18, 19, and 20 are the results of matching by several kinds of methods.  Figure 21 gives the last panoramic image stitched by our method.

### 4.5. Experimental Observations and Discussion

Based on the four different experiments presented previously, a significant advantage of our method should be highlighted, that is, the accuracy of matching. According to the experimental results, we can draw the following conclusions.

1. From Figures 3–6, our method significantly outperforms both traditional method based on grayscale feature and method based on SIFT feature in precision for the matching. The performance of the method based on grayscale feature is so serious that it even cannot accomplish the last step. It may be that the grayscale feature will change largely as the resolution changes.
2. From Figures 4 and 5, it can be demonstrated that the SIFT feature is robust to variance of resolution to some extent. It may be that the SIFT feature describes the local path of image. And our method outperforms the method based on the SIFT, since it extracts feature by describing the line in the image by SIFT feature.
3. From Figures 8–10, both SIFT and our method can obtain good result, as SIFT feature is invariant to rotation. And the grayscale is sensitive to rotation.
4. From Figures 13–15, both SIFT and our method can obtain good results, as SIFT feature is invariant to different light conditions. And the grayscale is sensitive to uneven illumination.
5. From Figures 9, 10, 14, and 15, our method has a higher accuracy, it may be that our method includes more information and the statistical voting strategy could acquire more accurate matching pairs.
6. From Figures 18–21, only the method proposed in the paper can obtain a good performance, as it could describe the lines of images. And the mean to describe the line is robust to object scaling. The methods based on the grayscale and SIFT cannot obtain good matching results; they even cannot accomplish the last stitching step.

In summary, the method proposed in this paper has a remarkable performance in image matching, since it is robust to difference of resolution, image scaling, rotation, and lighting.

## 5. Conclusions 

This paper proposed a new image mosaic method based on SIFT feature of directed line segment. This method has strong robustness for resolution, rotation, lighting, and scaling. The line-based description method proposed here has much robustness for image rotation and scaling; the description of directed line segment with SIFT feature can better avoid uneven lighting; and rough matching on the basis of statistical voting can acquire more accurate matching pairs and improve the quality of image mosaic.

## Figures and Tables

**Procedure 1:**
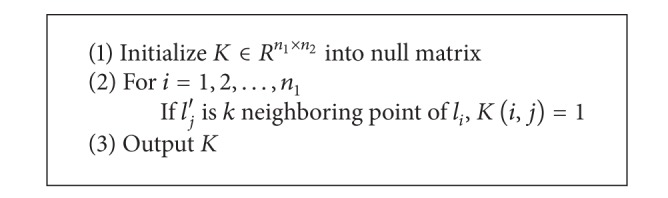
Method 1: computation procedure of matrix *K*.

**Procedure 2:**
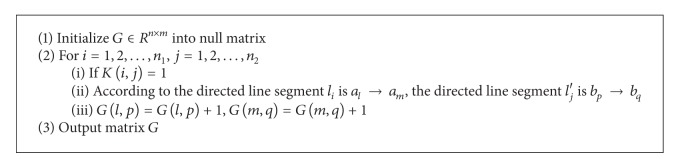
Method 2: computation procedure of matrix *G*.
